# Supplementary material for: Quantitative imaging reveals real-time Pou5f3–Nanog complexes driving dorsoventral mesendoderm patterning in zebrafish
Source: eLife. 2016 Sep 29;5:e11475. doi: 10.7554/eLife.11475 (PMC5042653; doi:10.7554/eLife.11475)
Supplement: Figure 1—source data 1. — Values for concentration and diffusion parameters were derived from the analysis of FCS data with the ACFs fit by two-component anomalous diffusion model. D1, D2: Diffusion coefficients of the fast and slow diffusion components, respectively. F2: Slow component fraction. α1, α2: anomalous parameters of the fast and slow diffusion components, respectively. Values represent mean ± SEM of data from three to five independent experiments (n represents the number of cell nuclei from 10 to 15 embryos; ****p<0.0001; **p<0.01; *p<0.05). Details of the rescue and the FCS analyses are shown in Figure 1—figure supplements 1–3 and Material and methods. DOI: http://dx.doi.org/10.7554/eLife.11475.004 [file elife-11475-fig1-data1.docx]

**Figure 1- source data 1 | Quantification of GFP-Oct4 concentration and activity in zebrafish rescued and non-rescued embryos**. Values for concentration and diffusion parameters were derived from the analysis of FCS data with the ACFs fit by two-component anomalous diffusion model. D_1_, D_2_: Diffusion coefficients of the fast and slow diffusion components, respectively. F_2_: Slow component fraction. α_1_, α_2_: anomalous parameters of the fast and slow diffusion components, respectively. Values represent mean ± SEM of data from three to five independent experiments (*n* represents the number of cell nuclei from 10–15 embryos; *****p*<0.0001; ***p*<0.01; **p*<0.05). Details of the rescue and the FCS analyses are shown in Figure 1 -supplement figure 1, Figure 1 -supplement figure 2, Figure 1 -supplement figure 3 and Material and Methods.

| **Stage** |  | **Concentration (nM)** | **D_1_**  **(μm^2^/s)** | **D_2_**  **(μm^2^/s)** | **F_2_**  **(DNA-bound Fraction)** | **α_1_** | **α_2_** | **n** |
| --- | --- | --- | --- | --- | --- | --- | --- | --- |
| **Oblong**  **(3.5 hpf)** | **GFP-Oct4 rescued**  **(10 pg)** | 32.22 ± 2.60** | 10.40 ± 0.04 | 0.66 ± 0.67* | 0.28 ± 0.01 | 0.99 ± 0.04 | 1.15 ± 0.03 | 56 |
|  | **GFP-Oct4 rescued**  **(20 pg)** | 44.39 ± 1.54 | 10.40 ± 0.04 | 0.91 ± 0.78 | 0.27 ± 0.01 | 0.77 ± 0.02 | 1.12 ± 0.02 | 125 |
|  | **GFP-Oct4**  **non-rescued**  **(20 pg)** | 43.9 0± 18.30 | 10.40 ± 0.04 | 0.65 ± 0.53* | 0.19 ± 0.08**** | 0.77 ± 0.02 | 1.07 ± 0.01 | 40 |
|  | **GFP-Oct4ΔD**  **non-rescued**  **(20 pg)** | 55.64 ± 14.86 | 10.40 ± 0.04 | 0.47 ± 0.22** | 0.12 ± 0.01**** | 0.94 ± 0.03 | 1.08 ± 0.04 | 39 |
|  | **GFP-Oct4 rescued**  **(40 pg)** | 83.23 ± 13.23** | 10.40 ± 0.04 | 0.95 ± 0.77 | 0.29 ± 0.03 | 0.92 ± 0.05 | 1.13 ± 0.05 | 42 |
| **60% epiboly**  **(7 hpf)** | **GFP-Oct4 rescued**  **(10pg)** | 21.25 ± 3.34 | 10.40 ± 0.04 | 0.50 ± 0.28 | 0.12 ± 0.04 | 0.83 ± 0.06 | 1.04 ± 0.03 | 38 |
|  | **GFP-Oct4 rescued**  **(20pg)** | 19.92 ± 0.55 | 10.40 ± 0.04 | 0.67 ± 0.46 | 0.12 ± 0.07 | 0.84 ± 0.02 | 1.11 ± 0.03 | 153 |
|  | **GFP-Oct4 rescued**  **(40pg)** | 21.05 ± 0.78 | 10.40 ± 0.04 | 0.41 ± 0.29 | 0.15 ± 0.01 | 0.88 ± 0.02 | 1.07 ± 0.02 | 38 |
