# Supplementary material for: Quantitative imaging reveals real-time Pou5f3–Nanog complexes driving dorsoventral mesendoderm patterning in zebrafish
Source: eLife. 2016 Sep 29;5:e11475. doi: 10.7554/eLife.11475 (PMC5042653; doi:10.7554/eLife.11475)
Supplement: Figure 2—source data 1. — Diffusion parameters values were derived from analysis of FCS data with the ACFs fit by two-component anomalous diffusion model. D1, D2: Diffusion coefficients of the fast and slow diffusion components, respectively. F2: Slow component fraction. α1, α2: anomalous parameters of the fast and slow diffusion components, respectively. ME: mesendoderm. EC: ectoderm. Values represent mean ± SEM from three to five independent experiments (n represents the number of cell nuclei from 10 to 15 embryos; ****p<0.0001; ***p<0.001). Details of the MOs and FCS analyses are shown in Figure 2—figure supplements 1,3 and Material and methods. DOI: http://dx.doi.org/10.7554/eLife.11475.009 [file elife-11475-fig2-data1.docx]

**Figure 2 –source data 1 | Quantification of GFP-Oct4 and GFP-Nanog activity in mesendoderm and ectoderm of *wt* and morphant zebrafish embryos**. Diffusion parameters values were derived from analysis of FCS data with the ACFs fit by two-component anomalous diffusion model. D_1_, D_2_: Diffusion coefficients of the fast and slow diffusion components, respectively. F_2_: Slow component fraction. α_1_, α_2_: anomalous parameters of the fast and slow diffusion components, respectively. ME: mesendoderm. EC: ectoderm. Values represent mean ± SEM from three to five independent experiments (*n* represents the number of cell nuclei from 10–15 embryos; *****p*<0.0001; ****p*<0.001). Details of the MOs and FCS analyses are shown in Figure 2 -figure supplement 1, Figure 2 -figure supplement 3 and Material and Methods.

|  | **D_1_**  **(μm^2^/s)** | **D_2_**  **(μm^2^/s)** | **F_2_**  **(DNA-bound**  **Fraction)** | **α_1_** | **α_2_** | **n** |
| --- | --- | --- | --- | --- | --- | --- |
| **GFP-Oct4 in ME** | 10.40 ± 0.04 | 0.81 ± 0.05 | 0.27 ± 0.01 | 0.96 ± 0.01 | 1.12 ± 0.02 | 111 |
| **GFP-Oct4 in EC** | 10.40 ± 0.04 | 0.80 ± 0.05 | 0.19 ± 0.01**** | 0.91 ± 0.02 | 1.04 ± 0.02 | 75 |
| **GFP-Oct4 in ME in *nanog* MO** | 10.40 ± 0.04 | 0.84 ± 0.04 | 0.22 ± 0.01*** | 0.96 ± 0.01 | 1.10 ± 0.02 | 167 |
| **GFP-Nanog in ME** | 14.80 ± 0.02 | 0.57 ± 0.04 | 0.21 ± 0.01 | 0.99 ± 0.02 | 1.07 ± 0.01 | 113 |
| **GFP-Nanog in EC** | 14.80 ± 0.02 | 0.56 ± 0.03 | 0.23 ± 0.01 | 0.98 ± 0.03 | 1.06 ± 0.02 | 83 |
| **GFP-Nanog in ME in *pou5f3* MO** | 14.80 ± 0.02 | 0.66 ± 0.06 | 0.16 ± 0.01*** | 0.84 ± 0.19 | 1.06 ± 0.02 | 60 |
