# Supplementary material for: Quantitative imaging reveals real-time Pou5f3–Nanog complexes driving dorsoventral mesendoderm patterning in zebrafish
Source: eLife. 2016 Sep 29;5:e11475. doi: 10.7554/eLife.11475 (PMC5042653; doi:10.7554/eLife.11475)
Supplement: Figure 2—source data 2. — Diffusion parameters values were derived from analysis of FCCS data with the ACFs and CCF fit by two-component anomalous diffusion model. D1, D2: Diffusion coefficient of the fast and slow diffusion component, respectively. α1, α2: anomalous parameter of the fast and slow diffusion component, respectively. Kd: dissociation constant at equilibrium; values were obtained from the slopes of the fitted linear line when plotting the concentration of GFP-Nanog (CN) * concentration of mCherry-Oct4 (CO) versus the concentration of the proteins association (CNO). If the proteins are associated, there will be a linear line; in cases where no association exists, there is no linear relationship (N.L). Association: fraction of proteins diffusing together in the same complex. Details of the FCCS analysis are shown in Figure 2—figure supplement 4 and Materials and methods. ME: mesendoderm. EC: ectoderm. Values represent mean ± SEM from three to five independent experiments with n > 15. DOI: http://dx.doi.org/10.7554/eLife.11475.010 [file elife-11475-fig2-data2.docx]

|  | **D_1_**  **(μm^2^/s)** | **D_2_**  **(μm^2^/s)** | **α_1_** | **α_2_** | ***Kd***  **(nM)** | **Association** |
| --- | --- | --- | --- | --- | --- | --- |
| **GFP-mCherry** | -- | 1.64 ± 0.09 | -- | 1.03 ± 0.02 | 1.84 ± 0.16 | 0.78 ± 0.10 |
| **GFP** | 22.94 ± 1.67 | -- | 0.95 ± 0.4 | -- | N.L | -- |
| **mCherry** | 18.06 ± 0.93 | -- | 0.93 ± 0.05 | -- |  |  |
| **ME** |  |  |  |  |  |  |
| **GFP-Nanog** | 14.80 ± 0.02 | 0.52 ± 0.05 | 0.65 ± 0.03 | 1.20 ± 0.06 | 15.4 ± 1.6 | 0.46 ± 0.06 |
| **mCherry-Oct4** | 15.38 ± 0.91 | 0.84 ± 0.08 | 0.89 ± 0.04 | 1.05 ± 0.06 |  |  |
| **EC** |  |  |  |  |  |  |
| **GFP-Nanog** | 14.80 ± 0.02 | 0.53 ± 0.05 | 0.75 ± 0.04 | 1.08 ± 0.03 | 61.9 ± 7.5 | 0.07 ± 0.01 |
| **mCherry-Oct4** | 15.38 ± 0.91 | 0.81 ± 0.06 | 0.91 ± 0.04 | 1.09 ± 0.06 |  |  |

**Figure 2 -source data 2**  **|** **FCCS parameters of GFP-Nanog and mCherry-Oct4 in mesendoderm and ectoderm of blastula embryos (oblong stage; 3.5 hpf).** Diffusion parameters values were derived from analysis of FCCS data with the ACFs and CCF fit by 2-component anomalous diffusion model. D_1_, D_2_: Diffusion coefficient of the fast and slow diffusion component respectively. α_1_, α_2_: anomalous parameter of the fast and slow diffusion component respectively. *Kd*: dissociation constant at equilibrium; values were obtained from the slopes of the fitted linear line when plotting the concentration of GFP-Nanog (C_N_) * concentration of mCherry-Oct4 (C_O_) versus the concentration of the proteins association (C_NO_). If the proteins are associated, there will be a linear line; in cases where no association exists, there is no linear relationship (N.L). Association: fraction of proteins diffusing together in the same complex. Details of the FCCS analysis are shown in Figure 2 -figure supplement 4 and Methods. ME: mesendoderm. EC: ectoderm. Values represent mean ± SEM from three to five independent experiments with *n*>15.
