# Supplementary material for: Quantitative imaging reveals real-time Pou5f3–Nanog complexes driving dorsoventral mesendoderm patterning in zebrafish
Source: eLife. 2016 Sep 29;5:e11475. doi: 10.7554/eLife.11475 (PMC5042653; doi:10.7554/eLife.11475)
Supplement: Figure 3—source data 1. — Diffusion parameters were derived from analysis of FCCS data with the ACFs and CCF fit by two-component anomalous diffusion model. D1, D2: Diffusion coefficients of the fast and slow diffusion components, respectively. α1, α2: anomalous parameters of the fast and slow diffusion components, respectively. Kd: dissociation constant at equilibrium; values were obtained from the slopes of the fitted linear line when plotting the concentration of GFP-Nanog (CN) * concentration of mCherry-Oct4 (CO) versus the concentration of the proteins association (CNO). If the proteins are associated, there will be a linear line; in cases where no association exists, there is no linear relationship. Association: fraction of proteins diffusing together in the same complex. Details of the FCCS analysis are explained in the Materials and methods. V-ME: ventral mesendoderm; ME: lateral mesendoderm; D-ME: dorsal mesendoderm; V-EC: ventral ectoderm; D-EC: dorsal ectoderm. Values represent mean ± SEM from three to five independent experiments with n > 15. DOI: http://dx.doi.org/10.7554/eLife.11475.016 [file elife-11475-fig3-data1.docx]

|  | **D_1_**  **(μm^2^/s)** | **D_2_**  **(μm^2^/s)** | **α_1_** | **α_2_** | ***Kd***  **(nM)** | **Association** |
| --- | --- | --- | --- | --- | --- | --- |
| **V-ME** |  |  |  |  |  |  |
| **GFP-Nanog** | 14.80 ± 0.02 | 0.65 ± 0.07 | 0.87 ± 0.03 | 1.01 ± 0.04 | 5.4 ± 0.4 | 0.35 ± 0.05 |
| **mCherry-Oct4** | 15.38 ± 0.91 | 0.71 ± 0.11 | 0.90 ± 0.04 | 0.97 ± 0.03 |  |  |
| **L-ME** |  |  |  |  |  |  |
| **GFP-Nanog** | 14.80 ± 0.02 | 0.67 ± 0.05 | 0.73 ± 0.03 | 1.21 ± 0.05 | 11.1 ± 0.73 | 0.38 ± 0.02 |
| **mCherry-Oct4** | 15.38 ± 0.91 | 0.68 ± 0.05 | 1.00 ± 0.04 | 0.98 ± 0.03 |  |  |
| **D-ME** |  |  |  |  |  |  |
| **GFP-Nanog** | 14.80 ± 0.02 | 0.54 ± 0.04 | 0.87 ± 0.03 | 1.00 ± 0.03 | 36.8 ± 3.5 | 0.14 ± 0.03 |
| **mCherry-Oct4** | 15.38 ± 0.91 | 0.53 ± 0.05 | 0.93 ± 0.08 | 0.98 ± 0.04 |  |  |
| **VL-EC** |  |  |  |  |  |  |
| **GFP-Nanog** | 14.80 ± 0.02 | 0.72 ± 0.11 | 0.83 ± 0.03 | 1.06 ± 0.04 | 57.09 ± 7.06 | 0.19 ± 0.12 |
| **mCherry-Oct4** | 15.38 ± 0.91 | 0.57 ± 0.08 | 0.90 ± 0.03 | 1.20 ± 0.05 |  |  |
| **D-EC** |  |  |  |  |  |  |
| **GFP-Nanog** | 14.80 ± 0.02 | 0.35 ± 0.03 | 0.85 ± 0.03 | 1.16 ± 0.07 | 201 ± 49.4 | 0.19 ± 0.12 |
| **mCherry-Oct4** | 15.38 ± 0.91 | 0.40 ± 0.04 | 0.96 ± 0.05 | 1.20 ± 0.08 |  |  |

**Figure 3 -source data 1**  **| FCCS parameters of GFP-Nanog and mCherry-Oct4 in** **mesendoderm and ectoderm of gastrula embryos (50% epiboly; 5.7 hpf**). Diffusion parameters were derived from analysis of FCCS data with the ACFs and CCF fit by two-component anomalous diffusion model. D_1_, D_2_: Diffusion coefficients of the fast and slow diffusion components, respectively. α_1_, α_2_: anomalous parameters of the fast and slow diffusion components, respectively. *Kd*: dissociation constant at equilibrium; values were obtained from the slopes of the fitted linear line when plotting the concentration of GFP-Nanog (C_N_) * concentration of mCherry-Oct4 (C_O_) *versus* the concentration of the proteins association (C_NO_). If the proteins are associated, there will be a linear line; in cases where no association exists, there is no linear relationship. Association: fraction of proteins diffusing together in the same complex. Details of the FCCS analysis are explained in the Methods. V-ME: ventral mesendoderm; ME: lateral mesendoderm; D-ME: dorsal mesendoderm; V-EC: ventral ectoderm; D-EC: dorsal ectoderm. Values represent mean ± SEM from three to five independent experiments with *n*>15.
