# Supplementary material for: Quantitative imaging reveals real-time Pou5f3–Nanog complexes driving dorsoventral mesendoderm patterning in zebrafish
Source: eLife. 2016 Sep 29;5:e11475. doi: 10.7554/eLife.11475 (PMC5042653; doi:10.7554/eLife.11475)
Supplement: Figure 5—source data 1. — Diffusion parameters were derived from analysis of FCCS data with the ACFs and CCF fit by two-component anomalous diffusion model. D1, D2: Diffusion coefficients of the fast and slow diffusion components, respectively. α1, α2: anomalous parameters of the fast and slow diffusion components, respectively. Kd: dissociation constant at equilibrium; values were obtained from the slopes of the fitted linear line when plotting the concentration of GFP-Sox32 (CS) * concentration of mCherry-Nanog (CN) versus the concentration of the proteins association (CSN). If the proteins are associated, there will be a linear line; in cases where no association exists, there is no linear relationship. Association: fraction of proteins diffusing together in the same complex. Details of the FCCS analysis are explained in the Materials and methods. D-E: dorsal endoderm; V-E: ventral endoderm; Values represent mean ± SEM from three to five independent experiments with n > 15. DOI: http://dx.doi.org/10.7554/eLife.11475.022 [file elife-11475-fig5-data1.docx]

**Figure 5 -source data 1**  **| FCCS parameters of GFP-Sox32 and mCherry-Nanog in** **endoderm of gastrula embryos (50% epiboly; 5.7 hpf**). Diffusion parameters were derived from analysis of FCCS data with the ACFs and CCF fit by two-component anomalous diffusion model. D_1_, D_2_: Diffusion coefficients of the fast and slow diffusion components, respectively. α_1_, α_2_: anomalous parameters of the fast and slow diffusion components, respectively. *Kd*: dissociation constant at equilibrium; values were obtained from the slopes of the fitted linear line when plotting the concentration of GFP-Sox32 (C_S_) * concentration of mCherry-Nanog (C_N_) *versus* the concentration of the proteins association (C_SN_). If the proteins are associated, there will be a linear line; in cases where no association exists, there is no linear relationship. Association: fraction of proteins diffusing together in the same complex. Details of the FCCS analysis are explained in the Methods. D-E: dorsal endoderm; V-E: ventral endoderm; Values represent mean ± SEM from three to five independent experiments with *n*>15.

|  | **D_1_**  **(μm^2^/s)** | **D_2_**  **(μm^2^/s)** | **α_1_** | **α_2_** | ***Kd***  **(nM)** | **Association** |
| --- | --- | --- | --- | --- | --- | --- |
| **D-E (50% epi)** |  |  |  |  |  |  |
| **GFP-Sox32** | 12.43 ± 0.90 | 0.81 ± 0.08 | 0.94 ± 0.03 | 0.95 ± 0.02 | 15.29 ± 1.17 | 0.17 ± 0.04 |
| **mCherry-Nanog** | 9.07 ± 0.36 | 0.57 ± 0.03 | 0.91 ± 0.04 | 1.25 ± 0.09 |  |  |
| **V-E (60% epi)** |  |  |  |  |  |  |
| **GFP-Sox32** | 12.43 ± 0.90 | 0.72 ± 0.06 | 0.89 ± 0.03 | 0.97 ± 0.03 | 14.59 ± 1.10 | 0.15 ± 0.05 |
| **mCherry-Nanog** | 9.07 ± 0.36 | 0.64 ± 0.05 | 0.84 ± 0.04 | 1.10 ± 0.07 |  |  |
