# Supplementary material for: Quantitative imaging reveals real-time Pou5f3–Nanog complexes driving dorsoventral mesendoderm patterning in zebrafish
Source: eLife. 2016 Sep 29;5:e11475. doi: 10.7554/eLife.11475 (PMC5042653; doi:10.7554/eLife.11475)
Supplement: Supplementary file 1. — Forward and reverse primers used in the qRT-PCR experiments. See Figure 6 and Figure 4—figure supplement 1. DOI: http://dx.doi.org/10.7554/eLife.11475.027 [file elife-11475-supp1.docx]

**Supplementary File 1 | List of primers.** Forward and reverse primers used in the qRT-PCR experiments. See Figure 6 and Figure 4 –figure supplement 1.

| ***Gene*** | **primer** | **sequence** |
| --- | --- | --- |
| *nanog* | forward | TGTACCCGCAAGTGTCAGAG |
|  | reverse | AACCCCTTCTCGACTGCTC |
| *ntl*  *wnt8a*    *oct4/pou5f3*  *cyclinB1*  *mespa*  *nnr*  *tbx6*  *her1*  *foxh1*  *has2*  *papc*  *mxtx2*  *bon*  *actin*  *sox32*  *her5*  *bmp2b*  *bmp2a*  *bmp4* | forward | TATTGCAGTCACAGCATATCAGAAT |
|  | reverse  forward  reverse  forward  reverse | AAGCTGGAGTATCTCTCACAGTACG  CTGCAGTTATCCACGCATAAAG  CAGCAGCACTTATAGCATGCAC  CTCCGAGAACCCTCAGGATA  CGACTCTAGAGCAGAACGGACT |
|  | forward  reverse  forward  reverse  forward  reverse  forward  reverse  forward  reverse  forward  reverse  forward  reverse  forward  reverse  forward  reverse  forward  reverse  forward  reverse  forward  reverse  forward  reverse  forward  reverse  forward  reverse  forward  reverse | CCACTACCCTCCCTCTCAGAT  TGCTGAAGAGTAGGGGTCCA  CCAGCTCAGCAGTTCATGTCT  AATTGTGCTGCTGGGAAAAC  TTTAAAGCTTCCATGCCTGTG  TGCAGTTTCCACAAGAGCAC  GATACCCAGAACTCTTCATCTGTCA  CTCATATGTGGTCTGTGTTCAGTCT  CGACTGCGAGAGATCAAGG  CATGGCATCTGGGGTCTC  GAACTCGCCAGAGAAGAAGC  ACAGGTTGTGTCGGACAGAGT  CCTATTTCAGAGAGTGGCTTTACAA  GAATGACATGAAGACCATGACTATG  AACCTTTTGAGCAGACATTTTTATG  AATCACACATGGTTTGATCTTCC    ACATGTGGACTGACTGCATTG  TGTTCTTTGGTGAAGCTGGTT  TTTTTCCAAACGCGGATATG  TCCTGAAGCGCATAATCTGA    GGCTACAGCTTCACCACCA  TGCTGATCCACATCTGCTG  CATCATCTGGACGAAAGAGGA  ACATTGCTTTCCATGTCTTGC    GGAGCAAAAAGACATGAGAAGG  TCTCAAGGTTTCTAGGCTTTGATT  GAT TCG CTG GAG ATG ATG  GTC TTT CTG TCC CAT ACC AA  ACTCCGTGAACGCAGAGCAG  TGGAGGTCAGGTTGAAGAGGAAC  GACATGACGGCAAAAGTCACC  TCGGCTAATGGAAAGGGACACTC |
